# N-linoleyltyrosine resisted the growth of non-small cell lung cancer cells via the regulation of CB_1_ and CB_2_ involvement of PI3K and ERK pathways

**DOI:** 10.3389/fphar.2023.1164367

**Published:** 2023-06-08

**Authors:** Yan Hu, Zhe Zhao, Yuan-Ting Liu, Ze-Cheng Xu, Jing-Yi Li, Zheng-Yu Yang, Rui-Wang, Yun-Qi Yang, Jia-Hui Zhang, Si-Yuan Qiu, Tao He, Yi-Ying Wu, Sha Liu

**Affiliations:** ^1^ The Second Affiliated Hospital of Chengdu Medical College (China National Nuclear Corporation 416 Hospital), Chengdu, Sichuan, China; ^2^ Department of Pharmacy, Study on the Structure-Specific Small Molecule Drug in Sichuan Province College Key Laboratory, Chengdu Medical College, Chengdu, Sichuan, China; ^3^ School of Laboratory Medicine, Chengdu Medical College, Chengdu, Sichuan, China; ^4^ School of Biological Sciences and Technology, Chengdu Medical College, Chengdu, Sichuan, China

**Keywords:** N-linoleyltyrosine, non-small cell lung cancer, phosphatidylinositol 3-kinase, extracellular signal-regulated kinase, c-Jun NH2-terminal kinase

## Abstract

**Background:** N-linoleyltyrosine (NITyr), one of the anandamide analogs, exerts activity via the endocannabinoid receptors (CB_1_ and CB_2_), which showed anti-tumor effects in various tumors. Therefore, we speculated that NITyr might show anti-non-small cell lung cancer (NSCLC) effects via the CB_1_ or CB_2_ receptor. The purpose of the investigation was to reveal the anti-tumor ability of NITyr on A549 cells and its mechanisms.

**Methods:** The viability of A549 cells was measured by MTT assay, and the cell cycle and apoptosis were both examined by flow cytometry; in addition, cell migration was tested by wound healing assay. Apoptosis-related markers were measured by immunofluorescence. The downstream signaling pathways (PI3K, ERK, and JNK) of CB_1_ or CB_2_ were examined through Western blotting. The expressions of CB_1_ and CB_2_ were detected by immunofluorescence. Finally, the AutoDock software was used to validate the binding affinity between the targets, such as CB_1_ and CB_2,_ with NITyr.

**Results:** We found that NITyr inhibited cell viability, hindered the cell cycle, resulted in apoptosis, and inhibited migration. The CB_1_ inhibitor, AM251, and the CB_2_ inhibitor, AM630, weakened the aforementioned phenomenon. The immunofluorescence assay suggested that NITyr upregulated the expression of CB_1_ and CB_2_. Western blot analysis indicated that NITyr upregulated the expression of p-ERK, downregulated the expression of p-PI3K, and did not affect p-JNK expression. In conclusion, NITyr showed a role in inhibiting NSCLC through the activation of CB_1_ and CB_2_ receptors involved in PI3K and ERK pathways.

## 1 Introduction

Non-small cell lung cancer (NSCLC) accounts for about 85% of lung cancers. Because of its high invasiveness and limited treatment options, the 5-year overall survival rate of NSCLC patients is low. In addition, the postoperative recurrence rate is very high (Siegel et al., 2021). In recent years, the endocannabinoid system (ECS) has become a vital target for treating different illnesses (Dando et al., 2013; Pellati et al., 2018; Hinz and Ramar, 2019; Ellert-Miklaszewska et al., 2021). Extensive studies on NSCLC *in vivo* and *in vitro* show that the ECS reduces neovascularization and migration of tumor cells by inhibiting the reproduction of cancer cells and resulting in apoptosis.

The ECS includes endocannabinoids (AEA and 2-AG), endocannabinoid receptor CBR (CB_1_ and CB_2_), and endocannabinoid metabolic enzymes (FAAH and MAGL), which are members of the serine hydrolase superfamily and hydrolyze AEA and 2-AG, respectively (Lu and Mackie, 2021). The effects of AEA and 2-AG are mainly mediated by CB_1_ and CB_2_. The CB_1_ receptor is mainly distributed in the central nervous systems (CNSs), and the expression of the CB_2_ receptor is mainly distributed in immune cells and at an average level in peripheral tissue (Ye et al., 2019). The relationship between ECS function and the biological processes of cancer is closely relevant, such as the growth, migration, and invasion of tumor cells (Preet et al., 2011). A large number of studies suggest that CB_1_ and CB_2_ proteins are overexpressed in tumor cells, such as those in NSCLC, gliomas, liver cancer, and pancreatic cancer. (Ayakannu et al., 2018; Kisková et al., 2019; Laezza et al., 2020; Milian et al., 2020; Shah et al., 2021). Meanwhile, AEA inhibits the viability of A549, SW620, and DLD1 cells, which are related to NSCLC and colon cancer, through the activation of CB_1_ and CB_2_ (Ravi et al., 2014; Kuc et al., 2012; Proto et al., 2012; Pasquariello et al., 2009). Therefore, AEA is considered to resist NSCLC and improve the survival rate of patients. Additionally, the tumor cells are also suppressed via inhibiting FAAH and MAGL, whose roles are to hydrolyze AEA and 2-AG (Slivicki et al., 2019). Moreover, the rapid inactivation of AEA reduces its anti-tumor activity (Willoughby et al., 1997). Generally, it is highly difficult to apply AEA in the clinic. Therefore, it is vital to develop AEA analogs.

In previous studies, our laboratory synthesized an AEA analog, N-linoleyltyrosine (NITyr). NITyr prevented transient cerebral ischemia in gerbils by regulating the PI3K/Akt signaling pathway involvement of the CB_2_ receptor (Cheng et al., 2019). At the same time, NITyr exerted neuroprotective effects in the regulation of autophagy via a cannabinoid receptor *in vitro* and *in vivo* (Liu et al., 2020; Long et al., 2021). In summary, NITyr exerted benign pharmacological activities through the ECS, and the ECS participated in the processes of tumor formation (de Melo Reis et al., 2021). Based on the above, it is speculated that NITyr might show anti-tumor effects through the ECS. In this experiment, the roles of NITyr on proliferation, apoptosis, and invasion in NSCLC were clarified, and the regulatory mechanism of the ECS on NITyr was further discussed.

## 2 Materials and methods

### 2.1 Materials

NITyr, obtained from our laboratory, as previously reported (Cheng et al., 2019). Caspase-3 (1: 500), caspase-9 (1: 500), Bax (1: 500), Bcl-2 (1: 500), ERK1/2 (1: 5,000), phospho-ERK1/2 (1: 5,000), and cyclin D1 (1: 2000) rabbit polyclonal antibodies (Abways, United States); PI 3 kinase p85 alpha (1: 500) and phospho-PI 3 kinase p85 alpha (1: 500) antibodies (Abways, United States); goat anti-rabbit IgG (H + L) conjugated to horseradish peroxidase (HRP) (1: 5,000) (Abways, United States); goat anti-rabbit IgG (H + L) conjugated to horseradish peroxidase (HRP) with Alexa Fluor 594 (1: 5,000) (Abways, United States); GAPDH (1: 10,000), CB_1_ (1: 2000) and JNK (1: 2000) rabbit polyclonal antibodies (Proteintech, Rosemont, IL, United States); phospho-JNK (Tyr185) recombinant (1: 5,000) antibody (Proteintech, Rosemont, IL, United States); CB_2_ antibody (1: 500) (Affinity Biosciences, Cincinnati, OH, United States); AM251 (200 μg/mL) (Sigma-Aldrich, Burlington, MA, United States); AM630 (200 μg/mL) (Sigma-Aldrich, Burlington, MA, United States); chemiluminescent HRP substrate (BMD, Merck Millipore, Burlington, MA, United States); and ethylene diamine tetraacetic acid (EDTA) (Beyotime Institute of Biotechnology, Inc., Jiangsu, China).

### 2.2 Cell culture and drug treatment

A549 cells were obtained from Shanghai Fuheng Biotechnology Co., Ltd. (Shanghai, China). A549 cells were cultivated with a cell culture medium that included RPMI-1640 medium, 10% fetal bovine serum (FBS), and 1% penicillin–streptomycin solution, placed in a standard 5% CO_2_ laboratory incubator at 37°C, and then sub-cultured on the third day. The experimental groups were divided as follows: i) control; ii) 10, 25, 50, 75, and 100 μg/mL NITyr; iii) NITyr + AM251 (200 μg/mL); iv) NITyr + AM630 (200 μg/mL); v) control + AM251 (200 μg/mL); vi) control + AM630 (200 μg/mL).

### 2.3 Cell viability assay

A549 cells (5 × 10^3^ cells/well) were grown in 96-well plates and then stored in an incubator at 37°C at a 5% CO_2_ for 1 day. Next, the cells were processed with various concentrations of NITyr (10, 25, 50, 75, and 100 μg/mL) for 12, 24, and 48 h. In total, 10 μL of 3- (4,5-dimethylthiazol-2-yl)-2,5-diphenyltetrazolium bromide (MTT) solution (5 mg/mL) was added to the 96-well plates, and the plates were placed in the aforementioned incubator for 4 h. Next, 100 μL of dimethyl sulfoxide (DMSO) was provided to dissolve formazan crystals in each well. Subsequently, the plates were sonicated for 10 min. Afterward, the absorption of each well at 560 nm was detected by using a VICTOR Nivo™ microplate reader (PerkinElmer, Inc., Waltham, MA, United States).

### 2.4 Flow cytometry analysis of apoptosis

A549 cells (6 × 10^4^ cells/well) were cultured into each well of the 6-well plates at a 5% CO_2_ incubator at 37°C for up to 1 day. Afterward, various concentrations of NITyr (10, 25, and 50 μg/mL) were added into the well to maintain A549 cells for up to 12, 24, and 48 h. Then, apoptotic cells were gathered into EP tubes and rinsed twice with cold phosphate-buffered saline (PBS). Additionally, the cells were centrifuged at 2000 rpm for 5 min at 4°C, then 200 μL 1× binding buffer, 2 μL Annexin V-FITC, and 2 μL propidium iodide (PI) was added into EP tubes in turn and stored for 8 min in the dark. Finally, the NovoCyte Quanteon flow cytometer (ACEA Biosciences-Agilent Technologies, Santa Clara, CA, United States) was used to detect all samples.

### 2.5 FCM analysis of the cell cycle

A549 cells were seeded into 6-well plates at a density of 6 × 10^4^ cells per well, and the aforementioned plates were incubated for 1 day at a 5% CO_2_ incubator at 37°C. Next, NITyr (10, 25, and 50 μg/mL) was provided to process A549 cells for 12, 24, and 48 h, respectively. Then, the cell supernatant was collected into EP tubes, and the remaining cells were flushed twice with 1× cold PBS, trypsinized, and collected into EP tubes. Subsequently, all cell suspensions aforementioned were centrifuged for 5 min at a speed of 2000 rpm and rinsed twice with cold PBS. Next, the cells were fixed with ice–cold 70% ethanol overnight at 4°C. Later, the cells were resuspended with PI Master Mix (5 μL PI and 20 μL RNase A in 275 µL PBS) after being centrifuged at 2000 rpm for 5 min and incubated for 15 min in the darkness. Eventually, the cell cycle was analyzed by flow cytometry (ACEA Biosciences-Agilent Technologies, Santa Clara, CA, United States) after cell filtration.

### 2.6 Wound healing assay

After A549 cells were transplanted at a density of 10^5^ cells/well in 6-well plates for 24 h, different concentrations of NITyr (10 and 25 μg/mL) were added into the well to maintain A549 cells. Subsequently, we drew two straight lines perpendicular to each other in every well to create the wound with a pipette tip. The pictures were photographed to measure the gap between the wound edges. Then, the cells were continuously cultured for 12 h, 24 h, and 48 h. In order to assess the distance between wound edges, the pictures were taken again, and the grayscale value of the aforementioned pictures was detected using ImageJ software.

### 2.7 Immunofluorescence assay

A549 cells were seeded on coverslips with a number of 5 × 10^5^ cells per well in 6-well plates and preserved for 1 day in an incubator at 37°C. Afterward, the cells were handled by NITyr (10, 25, and 50 μg/mL) for 24 h. The coverslips were rinsed with cold PBS three times for 5 min each. In total, 4% paraformaldehyde was subsequently provided to immobilize coverslips for 15 min. In addition, the cells were permeated with 0.5% Triton X-100 diluted with Tris-buffered saline with Tween-20 (TBST) for 20 min at ambient temperature. All coverslips were washed again and incubated for 45 min with the blocking solution containing 5% bovine serum albumin (BSA) at ambient temperature. Immediately after, the corresponding primary antibodies, which were formulated by 1× TBST containing 1% BSA, were used to incubate the cells overnight at 4°C. After being flushed for 3 min per time with 1× TBST (three times in total), the secondary antibodies conjugated with Alexa Fluor 594 were provided to incubate the cells for 60 min at ambient temperature in the dark. In addition, the nucleus of A549 cells was probed with 4′, 6-diamidino-2′-phenylindole (DAPI). Finally, all coverslips were washed for 5 min each time with 1× TBST (five times in all) and detected by using a BX63 fluorescence microscope (magnification ×40; Olympus Corporation, Tokyo, Japan).

### 2.8 Western blot analysis

After being seeded into culture dishes, the A549 cells at a density of 10^6^ cells per well were stored in a standard 5% CO_2_ laboratory incubator at 37°C for 1 day. The cells were administered based on the Western blot protocol after being supplied with 10, 25, and 50 μg/mL NITyr to treat cells (Liu et al., 2020). After the cells were washed three times with cold PBS, the prepared lysis buffer containing 1% protease inhibitor and 1% EDTA was added to the 6-well plates, which were placed in a thermal box filled with ice for up to half an hour. Afterward, the cells were scraped by a cell scraper, collected into EP tubes, and centrifuged at 12,000 × g at 4°C for 20 min. After centrifugation, the cell supernatant was gently transferred to newly pre-chilled EP tubes that were placed on ice. Depending on the protocol of the Easy II Protein Quantitative Kit (TransGen Biotech Co., Ltd., Beijing, China), the protein concentration was detected and adjusted, and then all samples were denatured at 100°C for 6 min. To separate the protein, 10% polyacrylamide gels were applied (Tris-HCl system). Next, the isolated protein was transblotted onto polyvinylidene difluoride (PVDF) membranes (Merck Millipore, Burlington, MA, United States), and 5% BSA was used to block the aforementioned membranes on a shaker at 37°C for 1 h. The next step was to incubate the aforementioned membranes with the relevant primary antibodies for at least one night at 4°C. The GAPDH rabbit polyclonal antibody, an internal control, was used to quantify target proteins. Subsequently, after being flushed for 5 min each time with 1× TBST (three times in total), all membranes were preserved with secondary HRP-conjugated antibodies for 60 min and then rinsed with 1× TBST as previously mentioned. To detect the protein bands, all membranes aforementioned were handled with the chemiluminescent HRP substrate. The ChemiDoc system was provided to capture the images of protein bands, and the grayscale value of protein bands was probed via ImageJ software 1.8.0.

### 2.9 Molecular docking verification

The AutoDock software was used to explore binding affinities between macromolecules and small molecules. The three-dimensional (3D) structure of NITyr was obtained from the PDB database (http://www.rcsb.org/). The protein was pre-processed using AutoDock tools by removing water molecules and adding hydrogen atoms. The 3D structures of the CB_1_ receptor agonist (AM11542, AEA) and CB_2_ receptor agonist (dronabinol, 2-AG) were obtained from the NCBI PubChem database (https://pubchem.ncbi.nlm.nih.gov/). All visualization results were analyzed using PyMOL software (https://www.pymol.org/).

### 2.10 Statistical analysis

All data analyses were performed using GraphPad Prism, and the results were presented as mean ± standard deviation (SD). Comparisons were evaluated by unpaired Student’s *t*-tests or ANOVA. When the *p*-value was less than 0.05, the outcome was considered to be significantly different.

## 3 Results

### 3.1 Effects of NITyr on the cell viability of A549 cells

**FIGURE 1:**
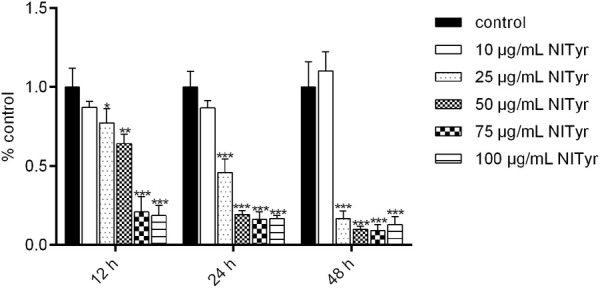
NITyr decreased the viability of A549 cells. MTT was used to detect the cell viability of A549 cells treated with 0, 10, 25, 50, 75, and 100 μg/mL NITyr for different times (12, 24, and 48 h). The above experiment was performed at least three times. *p < 0.05, **p < 0.01, and ***p < 0.001 compared to the control group.

As shown in Figure 1, compared to the control group, in the presence of NITyr at 12 h, the cell viability decreased to 77.33%, 64.33%, 21%, and 18.67% (*p* < 0.05, *p* < 0.01, and *p* < 0.001, respectively). After processing by NITyr for 24 h, the cell viability decreased to 46%, 19.33%, 16.33%, and 16.67% compared to the control group. After being processed by NITyr for up to 48 h, the cell viability decreased to 16.67%, 10%, 9.33%, and 13% compared to the control group (*p* < 0.05, *p* < 0.01, and *p* < 0.001, respectively). The aforementioned results indicated that NITyr inhibited the cell viability of A549.

### 3.2 Effects of NITyr on the cell cycle

**FIGURE 2:**
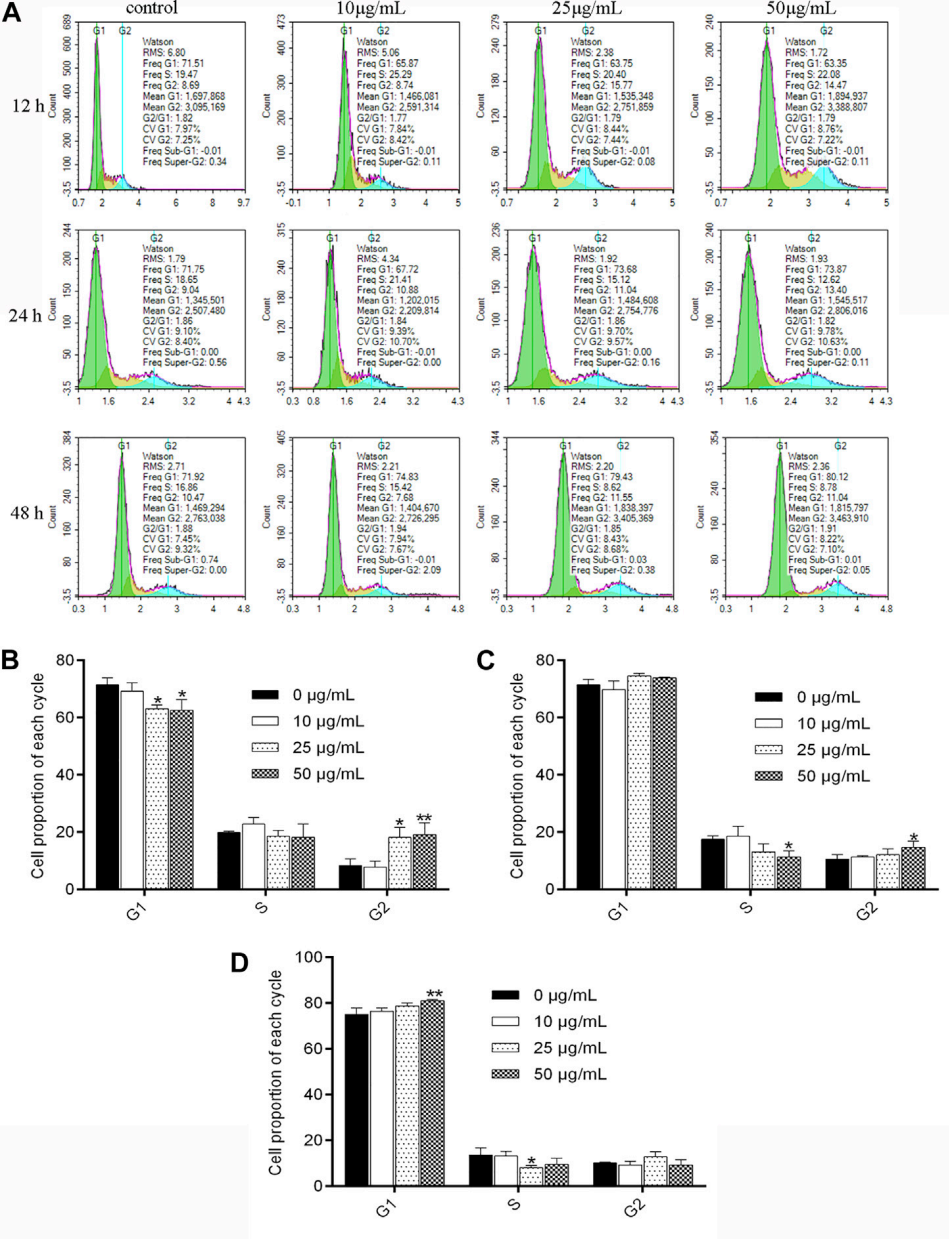
NITyr arrested the cell cycle of A549 cells. **(A)** Flow cytometry was performed to test the cell cycle of A549 cells treated with 0, 10, 25, and 50 μg/mL NITyr for different times, including 12, 24, and 48 h. **(B)** Statistical analysis of NITyr treatment for 12 h. **(C)** Statistical analysis of NITyr treatment for 24 h. **(D)** Statistical analysis of NITyr treatment for 48 h. The above experiment was conducted at least three times. *p < 0.05, **p < 0.01, and ***p < 0.001 compared to the control group.

As shown in Figure 2, after being treated with NITyr for 12 h, the ratio of cells increased from 71.5% to 63% and 62% (*p* < 0.05 both) in the G1 phase, the proportion of cells in the G2 phase increased from 8.377% to 18% and 19% (*p* < 0.05, *p* < 0.01), and the noticeable discrepancy was not tested in the S phase. After having dealt with NITyr for 24 h, the ratio of cells in the S phase decreased from 17.5% to 11% (*p* < 0.05), the proportion of cells in the G2 phase increased from 10.64% to 14% (*p* < 0.05), and an evident performance was observed in the G1 phase. In addition, after being handled by NITyr for 48 h, the ratio of cells in the G1 phase increased from 75.13% to 81 (*p* < 0.01), the ratio of cells in the S phase decreased from 13.64% to 8% (*p* < 0.05), and a notable discrepancy was observed in the G2 phase. The aforementioned results indicated that NITyr at various concentrations arrested the cell cycle at different phases.

### 3.3 Effects of NITyr on apoptosis of A549 cells

**FIGURE 3:**
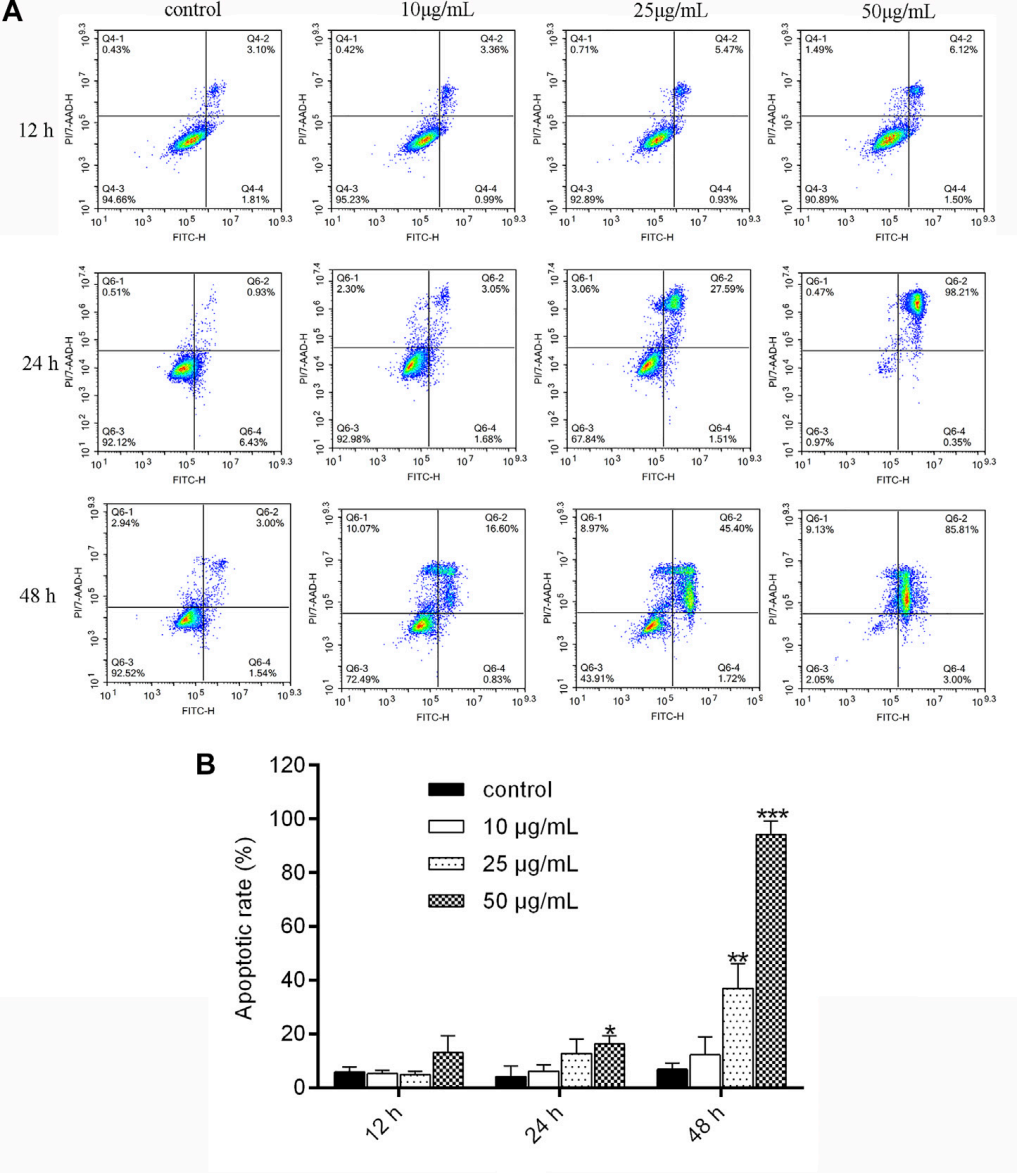
NITyr promoted A549 cell apoptosis. **(A)** Flow cytometry was performed to examine cell apoptosis at 12, 24, and 48 h after A549 cells were processed by NITyr (0, 10, 25, and 50 μg/mL). **(B)** Statistical analysis of flow cytometry. The above experiment was conducted at least three times. **p < 0.01 and ***p < 0.001 compared to the control group.

As shown in Figure 3, the apoptosis rate significantly increased from 6.377% to 18.33% (*p* < 0.01) after treatment with NITyr at 50 μg/mL when compared to the control group for 24 h. With the extension of time, the rate of cell apoptosis was notably increased at 48 h. The apoptosis rate increased from 6.943% to 37.02% and 94.19%, respectively, after 25 μg/mL and 50 μg/mL NITyr treatment (*p* < 0.01, *p* < 0.001) as compared with the control group. To sum up, NITyr promoted A549 cell apoptosis.

### 3.4 Effects of NITyr on the migration of A549 cells

**FIGURE 4:**
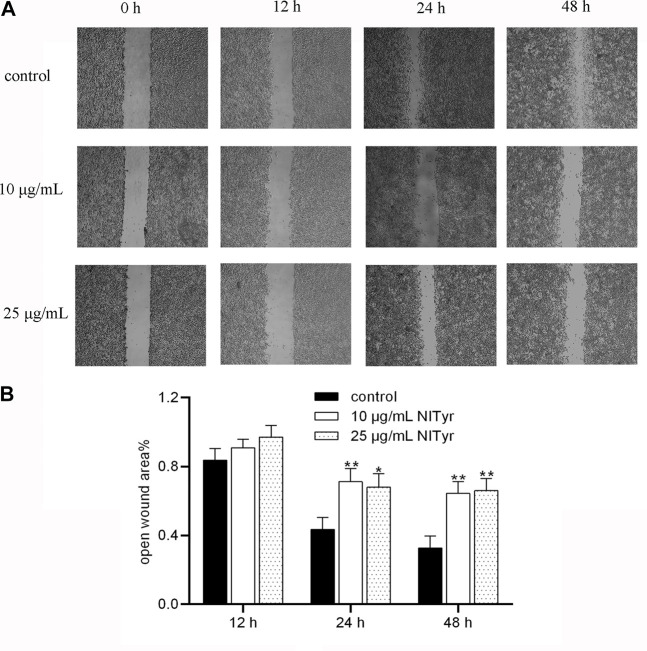
NITyr inhibited the migration ability of A549 cells. **(A)** The results of the control and experimental groups at 0 h, 12 h, 24 h, and 48 h were analyzed by using the wound healing assay. **(B)** Statistical analysis of the wound healing assay. The above experiment was conducted at least three times. *p < 0.05 and **p < 0.01 compared to the control group.

As shown in Figure 4, when the cells were administered with NITyr for 12 h, the open wound area in the NITyr groups was not significantly changed compared to the control group. As expected, in the presence of NITyr (10 μg/mL and 25 μg/mL) for 24 and 48 h, the open wound area was notably wider than in the control group (*p* < 0.05 and *p* < 0.01). In summary, NITyr showed an evident inhibition of the migration of A549 cells.

### 3.5 Effects of NITyr on apoptosis-related proteins

**FIGURE 5:**
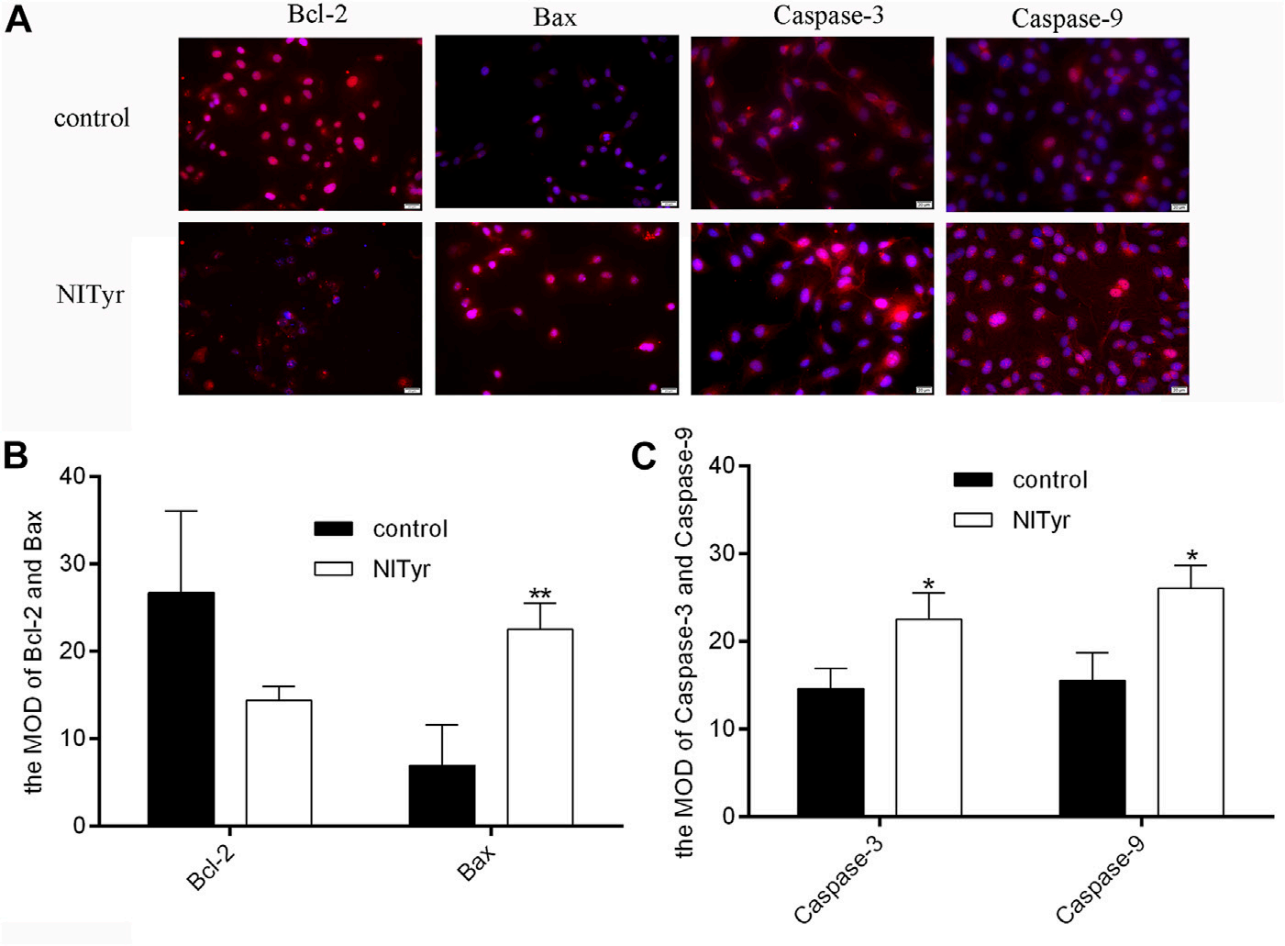
NITyr promoted the expression of apoptosis-related index. **(A)** The immunofluorescence test was used to assess the protein expressions of Bcl-2, Bax, caspase-3 and caspase-9. **(B)** The immunofluorescence images of Bax and Bcl-2 were statistically analyzed. **(C)** The immunofluorescence images of Caspase-3 and Caspase-9 were statistically analyzed. The above experiment was conducted at least three times. *p < 0.05 and **p < 0.01 compared to the control group.

As shown in Figure 5, the red fluorescence intensities of Bax, caspase-3, and caspase-9 were notably enhanced in the NITyr-treated groups as compared to the control group (*p* < 0.05, *p* < 0.01, and *p* < 0.01, respectively). Meanwhile, no prominent variation in the expression of Bcl-2 was investigated.

### 3.6 NITyr-regulated PI3K and ERK pathways

**FIGURE 6:**
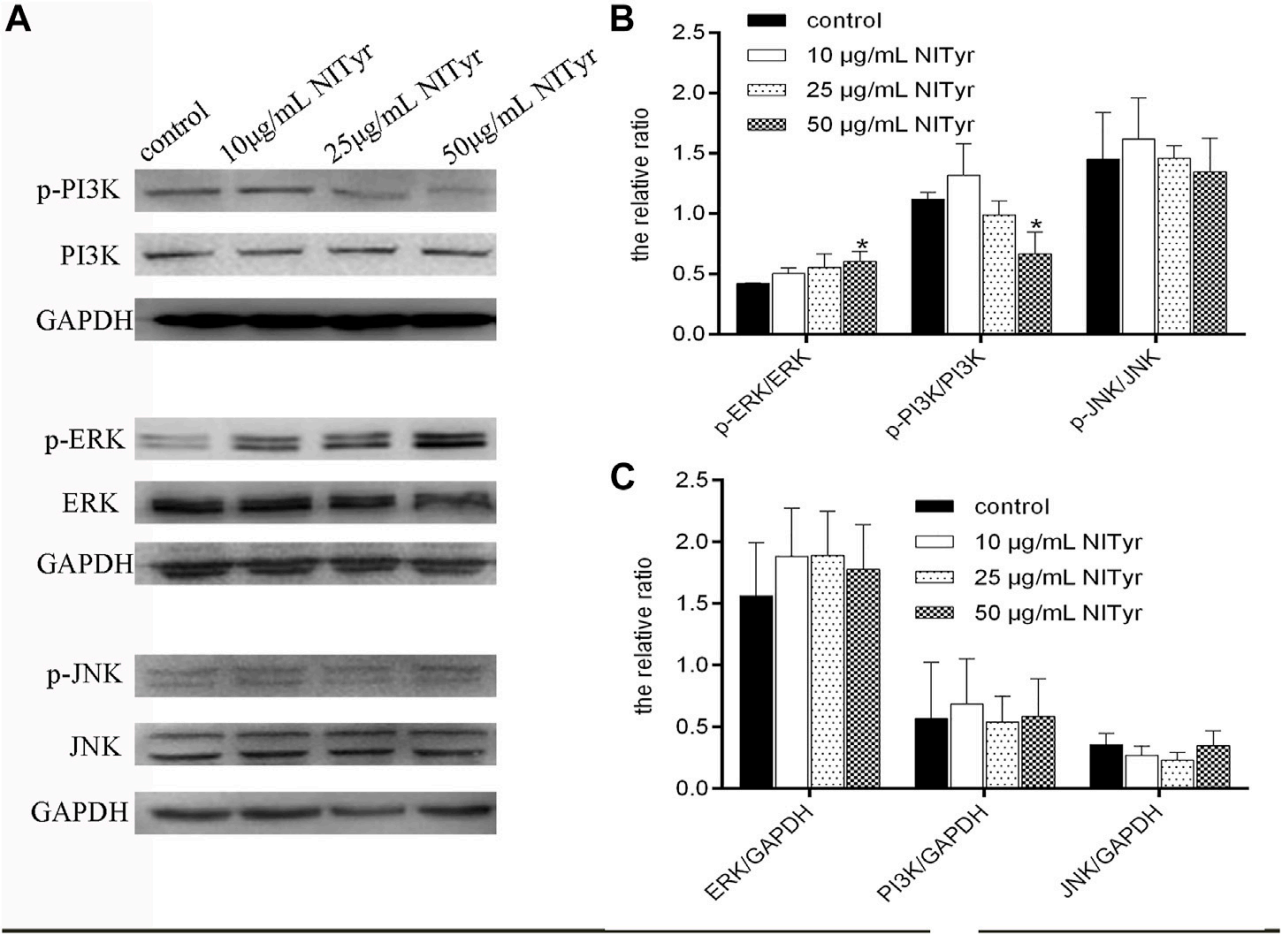
NITyr regulated the PI3K, ERK, and JNK pathways. **(A)** Western blotting was performed to test the expression of p-PI3K, PI3K, p-ERK, ERK, p-JNK, and JNK. **(B)** The relative ratios of p-PI3K/PI3K, p-ERK/ERK, p-JNK/JNK were statistically analyzed. **(C)** The relative ratios of ERK/GAPDH, PI3K/GAPDH, JNK/GAPDH were statistically analyzed. The above experiment was conducted at least three times. *p < 0.05, **p < 0.01, and ***p < 0.001 compared with the control group.

As shown in Figure 6, compared to the control group, the proportion of p-ERK/ERK increased obviously (*p* < 0.05), while that of p-PI3K/PI3K was downregulated (*p* < 0.05) in the NITyr groups. Meanwhile, in the presence of NITyr, no significance was detected in the ratio of p-JNK/JNK compared to the control group. Moreover, no obvious discrepancy was discovered in the non-phosphorylation expression of each pathway-related protein.

### 3.7 NITyr upregulated the protein expressions of CB_1_ and CB_2_ receptors

**FIGURE 7:**
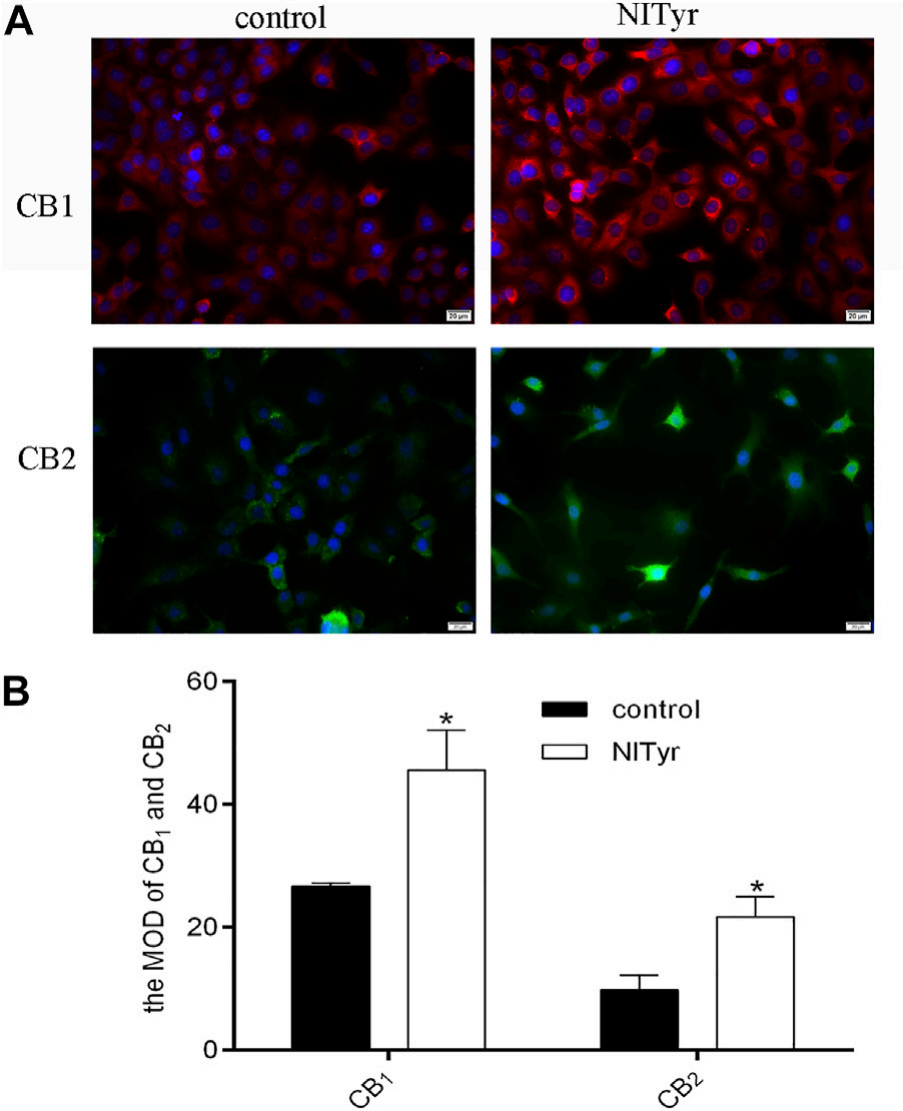
NITyr upregulated CB_1_ and CB_2_ expressions. **(A)** Immunofluorescence assay detected the expression of CB_1_ and CB_2_ proteins. **(B)** The immunofluorescence images were statistically analyzed. The above experiment was conducted at least three times. *p < 0.05 compared to the control group.

As shown in Figure 7, the fluorescence intensities were evidently enhanced in the NITyr-treated groups when compared to the control group, indicating that the expression of CB_1_ and CB_2_ proteins was upregulated.

### 3.8 AM251 and AM630 attenuated the effect of NITyr on cell viability and the migration of A549 cells

**FIGURE 8:**
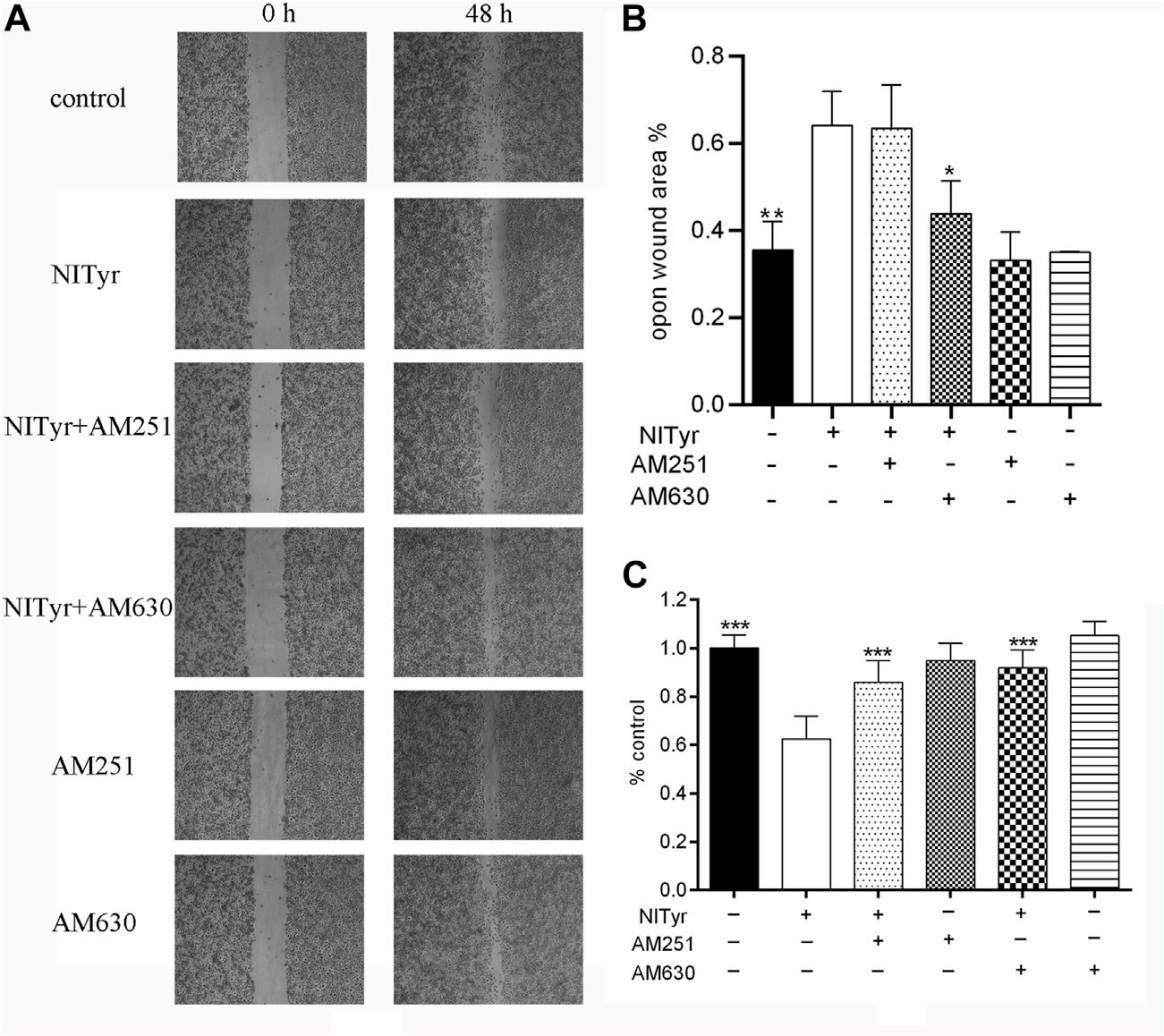
NITyr inhibited the ability of proliferation and migration by activating CB_1_ or CB_2_ receptors. **(A)** Scratch images were detected by using a microscope. **(B)** Statistical analysis of a cell-scratch experiment. **(C)** AM251 and AM630 weakened the effect of NITyr on cell viability. The above experiment was conducted at least three times. *p < 0.05, **p < 0.01, and ***p < 0.001 compared with the control group.

As shown in Figure 8, pre-treatment with AM251 and AM630 had no significance on the cell viability or migration of A549 cells compared with the control group. AM251 and AM630, combined with NITyr, exerted a weaker effect on cell viability. Furthermore, AM630 weakened the effect of NITyr on migration, while AM251 did not.

### 3.9 AM251 and AM630 attenuated the effect of NITyr on the bax expression

**FIGURE 9:**
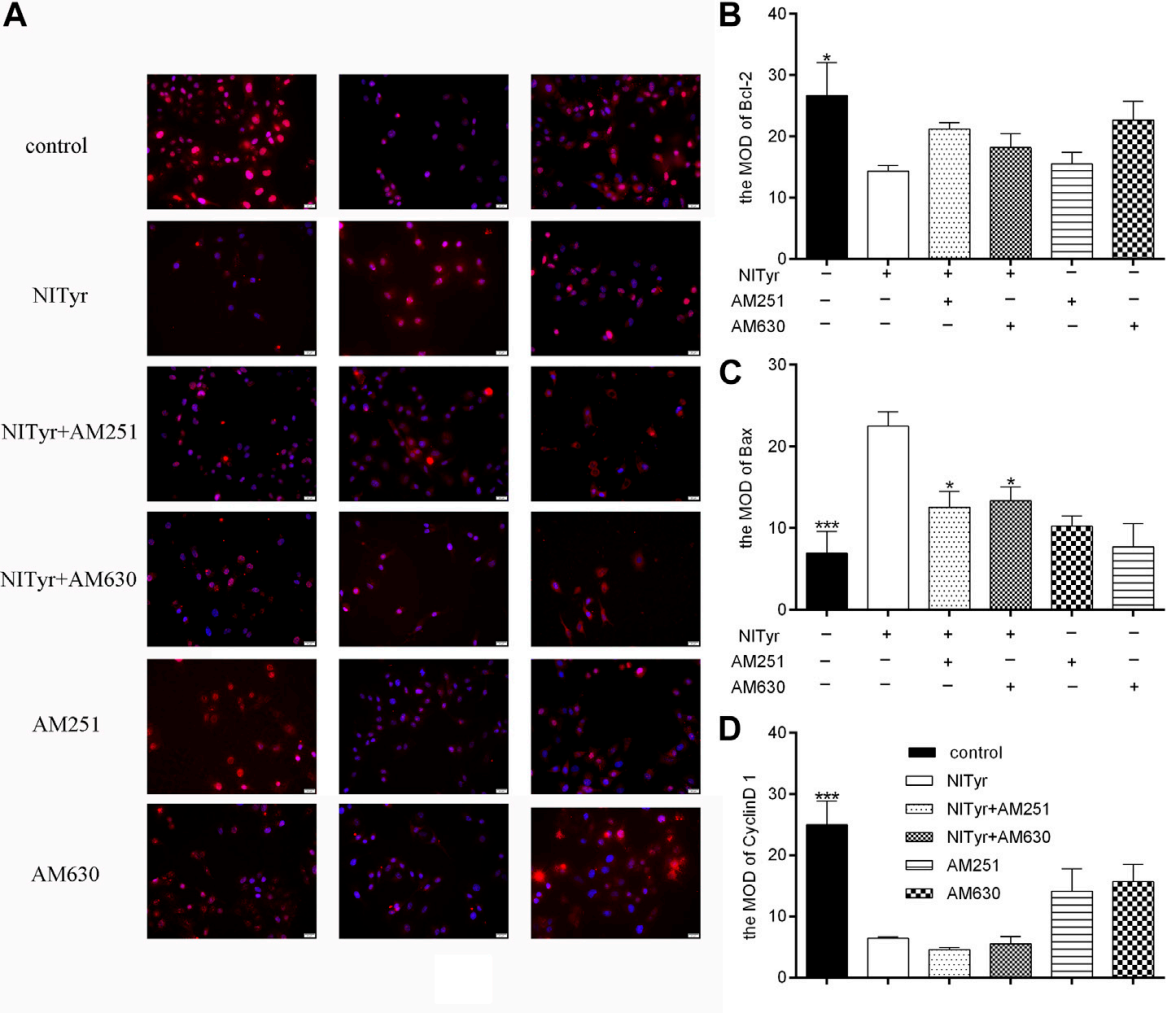
AM630 attenuated the effect of NITyr on Bax expression. **(A)** Immunofluorescence assay detected the expression of Bcl-2, Bax, and cyclin D1 proteins. **(B)** The immunofluorescence images of Bcl-2 were statistically analyzed **(C)** The immunofluorescence images of Bax were statistically analyzed. **(D)** The immunofluorescence images of cyclin D1 were statistically analyzed. The above experiment was conducted at least three times. *p < 0.05, **p < 0.01, and ***p < 0.001 compared with the control group.

As shown in Figure 9, pre-treatment with AM251 and AM630 had no significance on the expressions of Bcl-2, Bax, and cyclin D1 of A549 cells compared with the control group. AM251 or AM630 attenuated the effect of NITyr on Bax expression but not on the expression of Bcl-2 and cyclin D1.

### 3.10 Validation of molecular docking

**FIGURE 10:**
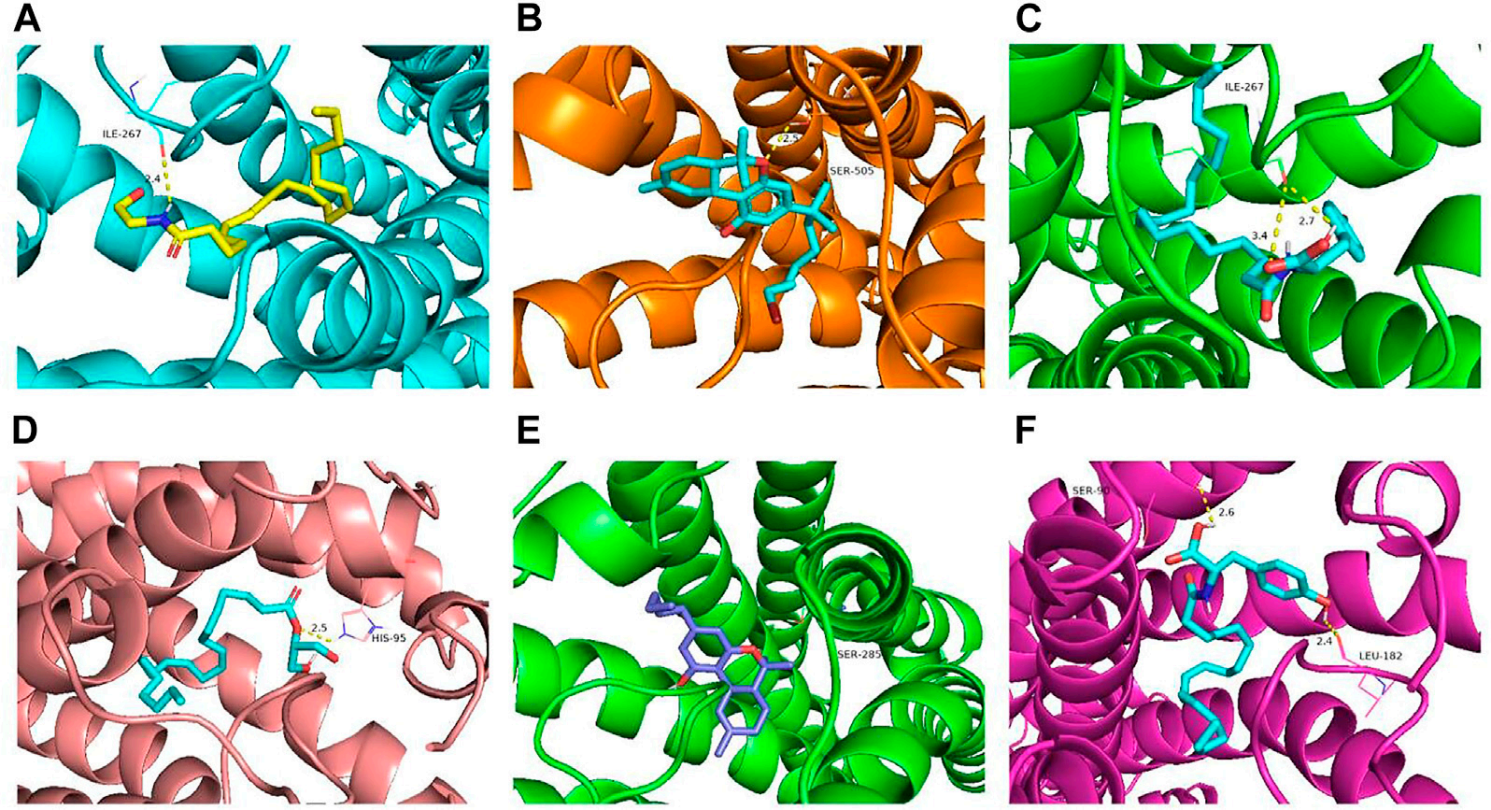
Molecular docking results of key targets (CB_1_ and CB_2_) with NITyr. **(A)** CB1 and AEA. **(B)** CB_1_ and AM11542. **(C)** CB_1_ and NITyr. **(D)** CB_2_ and 2-AG. **(E)** CB_2_ and dronabinol. **(F)** CB_2_ and NITyr.

AutoDock Vina molecular docking will obtain the score of each docking. The lower the docking score, the stronger the binding affinity with the target protein. These scores were compared with those of the positive drugs to predict potential targets. The docking scores of NITyr with CB_1_ and CB_2_ are shown in Table 1. As shown in Figure 10, All visualization results were analyzed using PyMOL software.

**TABLE 1:** Molecular docking scores.

Protein	PDB code	Ligand	Score (kcal/mol)
CB_2_	6kpc	Dronabinol	−11.5
		NITyr	−9.9
		2-AG	−8.8
CB_1_	7v3z	AM11542	−11.3
		NITyr	−9.9
		AEA	−9.1

## 4 Discussion

Numerous studies indicated that endocannabinoid analogs inhibited the multiplication, apoptosis, invasion, metastasis, and angiogenesis of cancers (Huang et al., 2021; Khodadadi et al., 2021; Mangal et al., 2021; Volmar et al., 2021; Wang et al., 2021). In addition, endocannabinoid analogs mainly acted on damaged tissues, but less on normal tissues, indicating they owned high selectivity (Meccariello R. 2020). However, the rapid metabolic deactivation of endocannabinoids limited the clinical application. Thus, the development of AEA analogs showed benign application prospects. In previous studies, NITyr, as an endocannabinoid analog, protected PC12 cells from oxidative damage via CB_1_ receptor regulation, prevented neurons against Aβ_1–40_-mediated cell toxicity through autophagy related to the CB_2_/AMPK/mTOR/ULK1 pathway (Zhou et al., 2022), and exerted neuroprotective activities in APP/PS1 transgenic mice through cannabinoid receptor-induced autophagy, indicating the neuroprotective activities of NITyr were related to endocannabinoid signal pathways. Furthermore, the intervention of the CB_1/2_ receptor improved the anti-tumorigenic effects in NSCLC (). In summary, it is speculated that NITyr also resisted NSCLC.

In the study, A549 cells were taken as the research object, and when the concentration of NITyr was 25 μg/mL, the cell viability was rapidly inhibited, and the inhibition rate was up to approximately 90%, indicating that NITyr inhibited cell viability at low concentrations.

In cell cycle experiments, after being processed with NITyr for 12 h, the ratio of cells in the G1 phase decreased while the proportion of cells in the G2 phase increased, suggesting that the cell reproduction was hampered in the G2 stage; after being treated with NITyr for 24 h, the ratio of cells in the S period declined as the number of cells in the G2 period increased, showing that the cell cycle was arrested in the G2 stage; after having dealt with NITyr for 48 h, the cell proportion in the S stage reduced while the cell proportion in the G1 period increased, suggesting the cell cycle was deterred in the G1 period. The aforementioned results indicated that with the prolongation of treatment time, the arresting effect of NITyr on cells gradually transited from the G2 phase to the G1 phase, and the arresting ability was steadily enhanced. The aforementioned results demonstrated that NITyr did inhibit cell proliferation.

In the scratch test, as the concentration of NITyr exceeded 25 μg/mL or the action time of NITyr exceeded 48 h, numerous cells died, and the phenomenon of cell migration could not apparently be observed; thus, the effects of NITyr within 48 h were observed. The scratch test simulated the processes of migration of A549 cells, and we analyzed the scratch width of cells to assess the migration of cells (Yi et al., 2021). The study demonstrated that NITyr remarkably prevented cell migration. Furthermore, the effects of NITyr at 12 h and 24 h were similar, indicating that its impact was not time-dependent. In the apoptosis experiment, NITyr mainly promoted the late apoptosis of cells, and the ratio of apoptotic cells gradually increased in a dose- and time-dependent manner, suggesting that NITyr could inhibit cell viability by promoting apoptosis.

Studies showed that CB_1_ and CB_2_ receptors regulate the growth, apoptosis, and migration of tumor cells (Preet et al., 2008; Elbaz et al., 2015; Mayor and Etienne-Manneville, 2016); thus, we further observed whether the effect of NITyr was related to CB_1_ and CB_2_ receptors. In the following experiment, we added CB_1_ and CB_2_ receptor inhibitors to observe the effect of NITyr on NSCLC. AM251 and AM630 inhibited the effect of NITyr on cell viability and migration. Moreover, AM630, but not AM251, was discovered to attenuate the expression of Bax induced by NITyr. The aforementioned results suggested that NITyr had anti-NSCLC effects by upregulating the expression of Bax mediated by CB_2_ receptors.

AEA analogs regulate PI3K, ERK, and JNK pathways via the activation of CB_1/2_ receptors (Sanchez et al., 2003; Ravi et al., 2014; Boyacioglu et al., 2021). PI3K possessed serine/threonine kinase activities. The p85 subunit of PI3K aggregated near the cell membrane when it received signals from the tyrosine kinase or G protein-coupled receptor, thereby promoting cell proliferation, inhibiting cell apoptosis, and ultimately promoting cell survival. The studies reported that the PI3K/Akt pathway was heavily implicated in tumorigenesis and the progression of NSCLC (Tan, 2020). ERK1/2 is an extracellular regulatory protein kinase that transmits signals from the surface receptor to the nucleus, thus mediating the reproduction of cells, discrepancy, and survival (Cagnol and Chambard, 2010). Moreover, excessive activation of ERK has been discovered in many tumor cells. Additionally, various compounds with anti-cancer properties have been indicated to lead to apoptosis in an ERK activation-dependent manner (Sugiura et al., 2021). JNK is a mitogen-activated protein kinase signaling pathway in mammalian cells. It not only plays the most crucial role in many physiological and pathological processes, such as cell proliferation, apoptosis, and stress but also plays a bigger role in the occurrence and development of tumors (Wu et al., 2019). The activation of the JNK pathway induces cell apoptosis in NSCLC (Tan et al., 2019).

In this study, the self-synthesized cannabinoid analog - NITyr - was used in an experimental group, and it significantly inhibited the proliferation and migration of A549 cells, promoted cell apoptosis, blocked the cell cycle, and upregulated the expression of apoptosis-related proteins (caspase-3, caspase-9, and Bax) and cannabinoid receptors (CB_1_ and CB_2_). In addition, NITyr markedly upregulated the proportion of p-ERK/ERK and notably downregulated that of p-PI3K/PI3K, suggesting that NITyr inhibited the growth or promoted apoptosis of NSCLC through the aforementioned pathways.

## Data Availability

The original contributions presented in the study are included in the article/Supplementary Materials; further inquiries can be directed to the corresponding author.

## References

[B1] Ayakannu T. Taylor A. H. Konje J. C. (2018). Cannabinoid receptor expression in estrogen-dependent and estrogen independent endometrial cancer. J. Recept Signal Transduct. Res. 38, 385–392. 10.1080/10799893.2018.1531890 30569804

[B2] Boyacıoğlu Ö. Bilgiç E. Varan C. Bilensoy E. Nemutlu E. Sevim D. (2021). ACPA decreases non-small cell lung cancer line growth through Akt/PI3K and JNK pathways *in vitro* . Cell Death Dis. 12 (1), 56. 10.1038/s41419-020-03274-3 33431819 PMC7801394

[B3] Cagnol S. Chambard J. (2010). ERK and cell death: Mechanisms of ERK-induced cell death—apoptosis, autophagy and senescence. FEBS J. 277, 2–21. 10.1111/j.1742-4658.2009.07366.x 19843174

[B4] Cheng L. Li J. Zhou Y. Zheng Q. Ming X. Liu S. (2019). N-linoleyltyrosine protects against transient cerebral ischemia in gerbil via CB2 receptor involvement in PI3K/akt signaling pathway. Biol. Pharm. Bull. 42, 1867–1876. 10.1248/bpb.b19-00394 31484847

[B5] Dando I. Donadelli M. Costanzo C. Dalla Pozza E. D'Alessandro A. Zolla L. (2013). Cannabinoids inhibit energetic metabolism and induce AMPK-dependent autophagy in pancreatic cancer cells. Cell Death Dis. 4 (6), e664. 10.1038/cddis.2013.151 23764845 PMC3698539

[B6] de Melo Reis R. A. Isaac A. R. Freitas H. R. de Almeida M. M. Schuck P. F. Ferreira G. C. (2021). Quality of life and a surveillant endocannabinoid system. Front. Neurosci. 15, 747229. 10.3389/fnins.2021.747229 34776851 PMC8581450

[B7] Elbaz M. Nasser M. W. Ravi J. Wani N. A. Ahirwar D. K. Zhao H. (2015). Modulation of the tumor microenvironment and inhibition of EGF/EGFR pathway: Novel anti-tumor mechanisms of cannabidiol in breast cancer. Mol. Oncol. 9 (4), 906–919. 10.1016/j.molonc.2014.12.010 25660577 PMC4387115

[B8] Ellert-Miklaszewska A. Ciechomska I. A. Kaminska B. (2021). Synthetic cannabinoids induce autophagy and mitochondrial apoptotic pathways in human glioblastoma cells independently of deficiency in *TP53* or *PTEN* tumor suppressors. Cancers (Basel) 13 (3), 419. 10.3390/cancers13030419 33499365 PMC7865605

[B9] Hinz B. Ramer R. (2019). Anti-tumour actions of cannabinoids. Br. J. Pharmacol. 176 (10), 1384–1394. 10.1111/bph.14426 30019449 PMC6487602

[B10] Huang T. Xu T. Wang Y. Zhou Y. Yu D. Wang Z. (2021). Cannabidiol inhibits human glioma by induction of lethal mitophagy through activating TRPV4. Autophagy 17 (11), 3592–3606. 10.1080/15548627.2021.1885203 33629929 PMC8632311

[B11] Khodadadi H. Salles E. L. Alptekin A. Mehrabian D. Rutkowski M. Arbab A. S. (2021). Inhalant cannabidiol inhibits glioblastoma progression through regulation of tumor microenvironment. Cannabis Cannabinoid Res. 10.1089/can.2021.0098 PMC10589502 34918964

[B12] Kisková T. Mungenast F. Suváková M. Jäger W. Thalhammer T. (2019). Future aspects for cannabinoids in breast cancer therapy. Int. J. Mol. Sci. 20 (7), 1673. 10.3390/ijms20071673 30987191 PMC6479799

[B13] Kuc C. Jenkins A. Van Dross R. T. (2012). Arachidonoyl ethanolamide (AEA)-induced apoptosis is mediated by J-series prostaglandins and is enhanced by fatty acid amide hydrolase (FAAH) blockade. Mol. Carcinog. 51 (2), 139–149. 10.1002/mc.20770 21432910 PMC3134573

[B14] Laezza C. Pagano C. Navarra G. Pastorino O. Proto M. C. Fiore D. (2020). The endocannabinoid system: A target for cancer treatment. Int. J. Mol. Sci. 21 (3), 747. 10.3390/ijms21030747 31979368 PMC7037210

[B15] Liu X. Wu Y. Zhou D. Xie Y. Zhou Y. Lu Y. (2020). N-Linoleyltyrosine protects PC12 cells against oxidative damage via autophagy: Possible involvement of CB1 receptor regulation. Int. J. Mol. Med. 46 (5), 1827–1837. 10.3892/ijmm.2020.4706 33000188 PMC7521587

[B16] Long C. M. Zheng Q. X. Zhou Y. Liu Y. T. Gong L. P. Zeng Y. C. (2021). N-linoleyltyrosine exerts neuroprotective effects in APP/PS1 transgenic mice via cannabinoid receptor-mediated autophagy. J. Pharmacol. Sci. 147 (4), 315–324. 10.1016/j.jphs.2021.08.008 34663513

[B17] Lu H. C. Mackie K. (2021). Review of the endocannabinoid system. Biol. Psychiatry Cogn. Neurosci. Neuroimaging 6 (6), 607–615. 10.1016/j.bpsc.2020.07.016 32980261 PMC7855189

[B18] Mangal N. Erridge S. Habib N. Sadanandam A. Reebye V. Sodergren M. H. (2021). Cannabinoids in the landscape of cancer. J. Cancer Res. Clin. Oncol. 147 (9), 2507–2534. 10.1007/s00432-021-03710-7 34259916 PMC8310855

[B19] Mayor R. Etienne-Manneville S. (2016). The front and rear of collective cell migration. Nat. Rev. Mol. Cell Biol. 17 (2), 97–109. 10.1038/nrm.2015.14 26726037

[B20] Meccariello R. (2020). Endocannabinoid system in Health and disease: Current situation and future perspectives. Int. J. Mol. Sci. 21 (10), 3549. 10.3390/ijms21103549 32443408 PMC7278997

[B21] Milian L. Mata M. Alcacer J. Oliver M. Sancho-Tello M. Martín de Llano J. J. (2020). Cannabinoid receptor expression in non-small cell lung cancer. Effectiveness of tetrahydrocannabinol and cannabidiol inhibiting cell proliferation and epithelial-mesenchymal transition *in vitro* . PLoS One 15, e0228909. 10.1371/journal.pone.0228909 32049991 PMC7015420

[B22] Pasquariello N. Catanzaro G. Marzano V. Amadio D. Barcaroli D. Oddi S. (2009). Characterization of the endocannabinoid system in human neuronal cells and proteomic analysis of anandamide-induced apoptosis. J. Biol. Chem. 284 (43), 29413–29426. 10.1074/jbc.M109.044412 19690173 PMC2785574

[B23] Pellati F. Borgonetti V. Brighenti V. Biagi M. Benvenuti S. Corsi L. (2018). *Cannabis sativa* L. And nonpsychoactive cannabinoids: Their chemistry and role against oxidative stress, inflammation, and cancer. Biomed. Res. Int. 2018, 1691428. 10.1155/2018/1691428 30627539 PMC6304621

[B24] Preet A. Ganju R. K. Groopman J. E. (2008). Delta9-tetrahydrocannabinol inhibits epithelial growth factor-induced lung cancer cell migration *in vitro* as well as its growth and metastasis *in vivo* . Oncogene 27 (3), 339–346. 10.1038/sj.onc.1210641 17621270

[B25] Preet A. Qamri Z. Nasser M. W. Prasad A. Shilo K. Zou X. (2011). Cannabinoid receptors, CB1 and CB2, as novel targets for inhibition of non-small cell lung cancer growth and metastasis. Cancer Prev. Res. (Phila). 4 (1), 65–75. 10.1158/1940-6207.CAPR-10-0181 21097714 PMC3025486

[B26] Proto M. C. Gazzerro P. Di Croce L. Santoro A. Malfitano A. M. Pisanti S. (2012). Interaction of endocannabinoid system and steroid hormones in the control of colon cancer cell growth. J. Cell Physiol. 227 (1), 250–258. 10.1002/jcp.22727 21412772

[B27] Ravi J. Sneh A. Shilo K. Nasser M. W. Ganju R. K. (2014). FAAH inhibition enhances anandamide mediated anti-tumorigenic effects in non-small cell lung cancer by downregulating the EGF/EGFR pathway. Oncotarget 5 (9), 2475–2486. 10.18632/oncotarget.1723 24811863 PMC4058020

[B28] Sanchez M. G. Ruiz-Llorente L. Sanchez A. M. Diaz-Laviada I. (2003). Activation of phosphoinositide 3-kinase/PKB pathway by CB(1) and CB(2) cannabinoid receptors expressed in prostate PC-3 cells. Involvement in Raf-1 stimulation and NGF induction. Cell Signal 15 (9), 851–859. 10.1016/s0898-6568(03)00036-6 12834810

[B29] Shah S. A. Gupta A. S. Kumar P. (2021). Emerging role of cannabinoids and synthetic cannabinoid receptor 1/cannabinoid receptor 2 receptor agonists in cancer treatment and chemotherapy-associated cancer management. J. Cancer Res. Ther. 17 (1), 1–9. 10.4103/jcrt.JCRT_488_18 33723124

[B30] Siegel R. L. Miller K. D. Fuchs H. E. Jemal A. (2021). Cancer statistics, 2021. CA Cancer J. Clin.;71(1):7–33. 10.3322/caac.21654 33433946

[B31] Slivicki R. A. Xu Z. Mali S. S. Hohmann A. G. (2019). Brain permeant and impermeant inhibitors of fatty-acid amide hydrolase suppress the development and maintenance of paclitaxel-induced neuropathic pain without producing tolerance or physical dependence *in vivo* and synergize with paclitaxel to reduce tumor cell line viability *in vitro* . Pharmacol. Res. 142, 267–282. 10.1016/j.phrs.2019.02.002 30739035 PMC6878658

[B32] Sugiura R. Satoh R. TakasakiERK T. (2021). Erk: A double-edged sword in cancer. ERK-dependent apoptosis as a potential therapeutic strategy for cancer. Cells 10 (10), 2509. 10.3390/cells10102509 34685488 PMC8533760

[B33] Tan A. C. (2020). Targeting the PI3K/Akt/mTOR pathway in non-small cell lung cancer (NSCLC). Thorac. Cancer 11 (3), 511–518. 10.1111/1759-7714.13328 31989769 PMC7049515

[B34] Tan B. Huang Y. Lan L. Zhang B. Ye L. Yan W. (2019). Bruceine D induces apoptosis in human non-small cell lung cancer cells through regulating JNK pathway. Biomed. Pharmacother. 117, 109089. 10.1016/j.biopha.2019.109089 31226632

[B35] Volmar M. N. M. Cheng J. Alenezi H. Richter S. Haug A. Hassan Z. (2021). Cannabidiol converts NF-κB into a tumor suppressor in glioblastoma with defined antioxidative properties. Neuro Oncol. 23 (11), 1898–1910. 10.1093/neuonc/noab095 33864076 PMC8563328

[B36] Wang K. Wang Q. Li Q. Zhang Z. Gao J. Fan C. (2021). Cannabinoid WIN55212-2 inhibits human glioma cell growth by triggering ROS-mediated signal pathways. Biomed. Res. Int. 2021, 6612592. 10.1155/2021/6612592 33977107 PMC8087470

[B37] Willoughby K. A. Moore S. F. Martin B. R. Ellis E. F. (1997). The biodisposition and metabolism of anandamide in mice. J. Pharmacol. Exp. Ther. 282, 243–247. 9223560

[B38] Wu Q. Wu W. Fu B. Shi L. Wang X. Kuca K. (2019). JNK signaling in cancer cell survival. Med. Res. Rev. 39 (6), 2082–2104. 10.1002/med.21574 30912203

[B39] Ye L. Cao Z. Wang W. Zhou N. (2019). New insights in cannabinoid receptor structure and signaling. Curr. Mol. Pharmacol. 12 (3), 239–248. 10.2174/1874467212666190215112036 30767756 PMC6864585

[B40] Yi S. Li Z. Wang X. Du T. Chu X. (2021). Circ_0001806 promotes the proliferation, migration and invasion of NSCLC cells through miR-1182/NOVA2 Axis. NOVA2 Axis 13, 3067–3077. 10.2147/CMAR.S290059 PMC8041606 33854376

[B41] Zhou Y. Li Z. X. Liu Y. T. Xu Z. C. Hu Y. Lv W. (2022). N-linoleyltyrosine protects neurons against Aβ1-40-induced cell toxicity via autophagy involving the CB2/AMPK/mTOR/ULK1 pathway. Brain Res. Bull. 188, 203–213. 10.1016/j.brainresbull.2022.08.002 35934162

